# Duration of Zearalenone Exposure Has Implications on Health Parameters of Lactating Cows

**DOI:** 10.3390/toxins16030116

**Published:** 2024-02-27

**Authors:** Raul Rivera-Chacon, Thomas Hartinger, Ezequias Castillo-Lopez, Claudia Lang, Felipe Penagos-Tabares, Rita Mühleder, Rana Muhammad Atif, Johannes Faas, Qendrim Zebeli, Sara Ricci

**Affiliations:** 1 Institute of Animal Nutrition and Functional Plant Compounds, Department for Farm Animals and Veterinary Public Health, University of Veterinary Medicine Vienna, Veterinärplatz 1, 1210 Vienna, Austria; thomas.hartinger@vetmeduni.ac.at (T.H.); ezequias.castillo-lopez@vetmeduni.ac.at (E.C.-L.); claudia.lang@vetmeduni.ac.at (C.L.); 11805328@students.vetmeduni.ac.at (R.M.); rana.atif@vetmeduni.ac.at (R.M.A.); qendrim.zebeli@vetmeduni.ac.at (Q.Z.); sara.ricci@vetmeduni.ac.at (S.R.); 2 Unit Nutritional Physiology, Institute of Physiology, Pathophysiology and Biophysics, Department of Bio-Medical Sciences, University of Veterinary Medicine Vienna, Veterinärplatz 1, 1210 Vienna, Austria; felipe.penagostabares@vetmeduni.ac.at; 3 dsm-firmenich, ANH R&D Center Tulln, 3430 Tulln, Austria; johannes.faas@dsm-firmenich.com

**Keywords:** mycotoxin, feed contamination, cattle, animal health, rumination, osmolality, milk

## Abstract

There is a limited research focus on evaluating the detrimental effects of prolonged zearalenone (ZEN) intake on dairy cows’ health under controlled conditions. This experiment was conducted to evaluate whether the length of exposure to a ZEN-contaminated total mixed ration (TMR) at a level of 9.45 mg per day can negatively influence animal health parameters, such as milk composition, rumen and fecal fermentation, and the chewing activity of lactating dairy cows. For this experiment, we used 18 lactating Simmental cows that were fed a diet of 60% forage and 40% concentrate (on dry matter basis) for 26 consecutive days. The first 4 days were for adaptation prior to the first sampling day (day 0). The sampling events took place on day 0 (baseline) without ZEN, followed by day 1, day 7, day 14, and day 21 (with toxin). Dry matter intake (DMI) and ruminating chews per minute increased on the third week of ZEN inclusion; meanwhile, ruminating, eating, and drinking times were not affected. Most milk composition variables were also unaffected. Rumen fluid osmolality increased on day 21 and total short-chain fatty acids (SCFA) of ruminal fluid decreased on day 7. Fecal SCFA increased on day 21 and the acetate-to-propionate ratio increased from day 1 onwards, showing the influence of toxin intake. Animal health parameters, like heart rate, respiratory rate, and body temperature, were negatively influenced by ZEN intake, all increasing consistently on days 4 and 6, 9 and 12, and 16 and 18, respectively. The liver enzyme glutamate dehydrogenase decreased in response to ZEN intake on day 7. A total daily ZEN intake at the level of 9.45 mg did not show detrimental effects on DMI. Nevertheless, certain health parameters were negatively affected, including body temperature, respiratory rate, and heart rate, starting from the 7th day of ZEN intake, with additional signs of possible loss of water balance on the last sampling day.

## 1. Introduction

Mycotoxins can be characterized as the natural toxicants that are most relevant to public health due to their widespread distribution in food and feed [1,2,3], and mycotoxin contamination can be worsened by climate change and droughts [4,5]. Within the group of mycotoxins, zearalenone (ZEN) is a resorcyclic lactone typically produced by various *Fusarium* species [6]. ZEN is also defined as a mycoestrogen due to its affinity for estrogen receptors, and thus can impair the reproduction of chronically exposed livestock [6,7,8]. Only over the last few decades has the natural occurrence of ZEN been reported. A study that quantified feed- and food-contaminated samples from 19 [9] and 100 [10] countries demonstrated a whole range of grain contamination of *Fusarium* mycotoxins, including wheat, barley, rice, corn, rye, oats, and their byproducts. This study found that ZEN occurred in 56% of the analyzed finished feeds (*n* = 19,171), presenting a maximum concentration of 9432 µg/kg, respectively. Additionally, research demonstrated that ZEN and its metabolite α-zearalanol (α-ZAL) were identified in milk samples from a range of countries, including China, Egypt, Hungary, and the UK, reaching up to 10.1 μg/kg of ZEN [7,11,12,13]. In terms of the European legislation, the guidance values recommended for ZEN in feedstuffs (considering a moisture content of 12%) range from 2000 μg/kg for cereal and cereal products to 3000 μg/kg for maize byproducts and 500 μg/kg for complementary and complete feedstuffs for ruminant species, including dairy cattle, goats, and sheep [14]. An American study [15] quantified losses due to Fusarium contamination, estimating them to be around tens of millions of dollars compared with a normal year.

Therefore, ZEN is relevant in cattle production systems. Considering that high-yielding dairy cows have a higher energy and protein demand than dry cows or heifers, and thus are more susceptible to metabolic problems [16], ZEN intake may have other detrimental effects on milk production. Different studies demonstrate that ruminants can degrade these metabolites to a certain degree; for instance, Kiessling et al. [17] showed that not only protozoa but also bacteria can turn ZEN into α-Zearalenol (α-ZOL) and to a lesser extent to β-Zearalenol (β-ZOL). Similarly, different in vitro studies demonstrated that rumen microbiota can convert 25–90% of ZEN into α-ZOL and to a lesser extent to β-ZOL [13,14,15], which was also confirmed in vivo [18]. Furthermore, α-ZOL is 60 times as potent as ZEN; meanwhile, β-ZOL is only 0.2 times as potent [19]. Bacterial strains, such as *Bacillus subtilis* and *Pseudomonas gessardii,* were demonstrated to neutralize ZEN into non-hazardous compounds [20]. In a study with goats, Dong et al. [21] confirmed that liver may induce a greater metabolization rate of these molecules than the rumen, but in terms of conversion per unit of mass, the whole gastrointestinal tract is comparably efficient.

Research in rumen fermentation has been conducted, proving that the changes in bacterial and protozoal metabolism caused by ZEN intake also determine changes in short-chain fatty acid (SCFA) fermentation. For instance, Hartinger et al. [22] quantified a decrease in total SCFA concentration and an increment in isobutyrate proportion one day after cows were exposed to 5.9 mg of ZEN. There are also inconsistent results in terms of rumen pH, ammonia and SCFA concentrations in the rumen after the inclusion of ZEN in the ration [23,24,25]. As an example, Keese et al. [26] reported an increase in valerate when cows were fed *Fusarium*-contaminated feed. To a lesser extent, damage to the gastrointestinal tract was reported to cause the death of epithelial cells in pigs and other species [27], or improve cell proliferation, increase colony formation and accelerate the cell migration of colon carcinoma in humans [28] exposed to ZEN. In terms of animal health response, multiple studies have been conducted over the years to evaluate different toxin concentrations. Noller et al. [29] reported that rations containing 100 ppb ZEN with 2.5 ppb deoxynivalenol (DON) caused a 8.1 kg loss of weight over 21 days in lactating cows. ZEN intake is also related to reduced DMI, vulva swelling, and reduced milk yield [30,31]. However, most of the reports focus on reproductive health parameters alone, and there is a lack of studies evaluating the effects of ZEN exposure on the systemic health of cattle. Under controlled conditions, there is limited information monitoring systemic animal health parameters. In this context, Hartinger et al. [22] reported that ZEN intake increased heart rate and body temperature following short-term exposure.

Most current mycotoxin research focuses on short-term and synergistic effects, predominantly in relation to reproductive parameters. Due to the lack of information related to animal response after a medium-length exposure to ZEN alone, we aimed to evaluate if a low dose of ZEN shows detrimental effects in terms of animal health parameters, liver enzymes, rumen fermentation, milk composition, and chewing activity. Our hypothesis states that contamination with 9.45 mg/day of ZEN in a 40% concentrate diet (DM basis) for 21 days would cause negative effects on health parameters in lactating cows. More specifically, we hypothesize the occurrence of liver burden, decreased DMI, and impaired rumination due to the duration of mycotoxin exposure.

## 2. Results

### 2.1. Feed Intake and Chewing Activity

Daily DMI increased as the trial came to an end, reaching the highest values on weeks 2 and 3 with increases of 7 and 6% compared with baseline (*p* < 0.01). ZEN did not influence the main chewing activity indicators, including ruminating time, eating time, ruminating chews per bolus, drinking time, drinking gulps, total chewing time, and chewing index. Ruminating chews per minute increased from 66.25 to 67.77 on baseline and week 3, respectively (*p* < 0.01) as shown in Table 1.

### 2.2. Rumen Fluid pH and Osmolality

**Figure 1:**
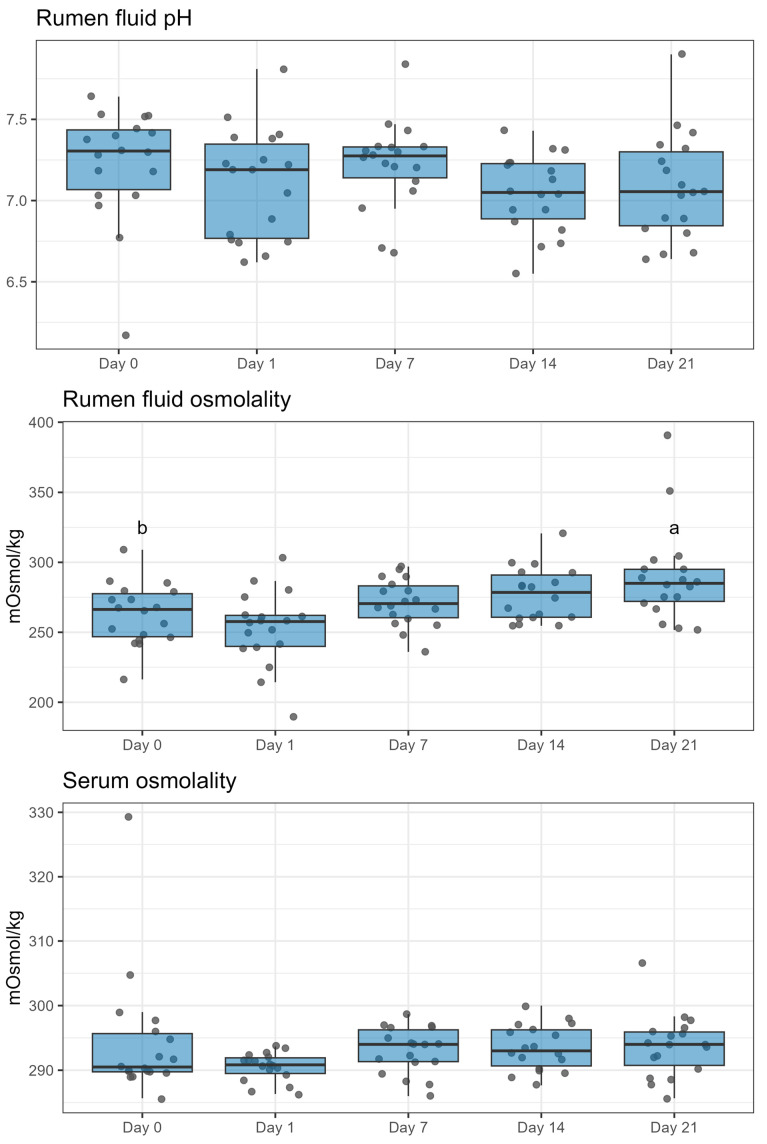
Effect of duration of exposure to ZEN (9.45 mg/day) on rumen fluid pH and osmolality and blood serum of lactating Simmental cows. Different letters (a, b) indicate a significant difference (*p* < 0.05) compared to day 0. The duration of exposure to ZEN had a significant effect on rumen osmolality (*p* < 0.01) but not on the other parameters.

Rumen fluid pH was not affected by the duration of ZEN exposure. Nevertheless, the osmolality of ruminal fluid increased from day 0 (baseline) to day 21 (*p* < 0.01). Similarly, this parameter increased on days 14 and 21 compared to day 1 with *p* < 0.05 and *p* < 0.01, respectively (Figure 1).

### 2.3. Rumen Fluid and Fecal SCFA

In terms of rumen fluid, total SCFA, which decreased on day 7 compared to day 0 (baseline) (*p* < 0.01), was also accompanied by decreasing caproate (*p* < 0.05) and a trend for decreasing isobutyrate (*p* = 0.07). Isobutyrate proportion decreased consistently on days 14 and 21 (*p* < 0.05). Caproate tended to decrease on day 14 (*p* = 0.08), and valerate tended to decrease on days 14 and 21 with *p* = 0.06 and *p* = 0.09, respectively. There were no changes in the major rumen fermentation acids nor isovalerate, heptanoate and the acetate-to-propionate ratio (Table 2). On the other hand, fecal fermentation revealed an initial increase in acetate complemented with a decrease in propionate, isobutyrate, and valerate and an increase in acetate-to-propionate ratio from day 1 onwards. These changes were replicated with a greater intensity with the addition of a decreased level of valerate on days 14 and 21 (*p* < 0.05) compared to day 0. Additionally, total SCFA increased on day 21 compared to day 0.

### 2.4. Milk Yield and Composition

Daily milk yield and composition parameters are indicated in Table 3. Milk yield, energy-corrected milk (ECM) and most of milk composition parameters, including fat, protein, lactose, fat–protein ratio and somatic cell count (SCC) were not affected by ZEN exposure. However, milk urea nitrogen (MUN) increased on day 7 (*p* < 0.05); similarly, compared to day 0, milk pH initially decreased on day 7 and increased on day 21 with *p* < 0.01 and *p* < 0.05, respectively.

### 2.5. Animal Health Parameters

**Figure 2:**
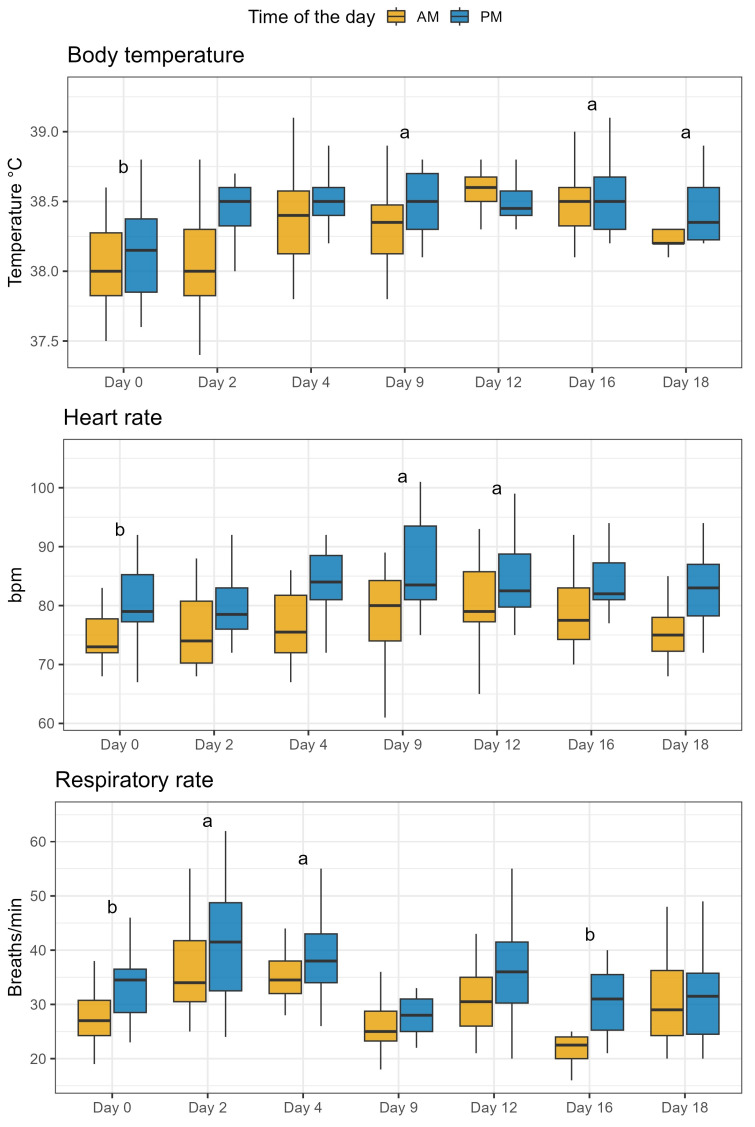
Effect of duration of exposure to ZEN (9.45 mg/day) on health parameters of lactating Simmental cows. Measurements were taken twice per day, after each feeding time in the morning (AM) and afternoon (PM). Different letters (a, b) indicate a significant difference (*p* < 0.05) compared to day 0. The duration of exposure to ZEN had a significant effect on all the health parameters (*p* < 0.01), while a significant difference between morning and afternoon measurements was found only for heart rate and respiratory rate (*p* < 0.01).

**Figure 3:**
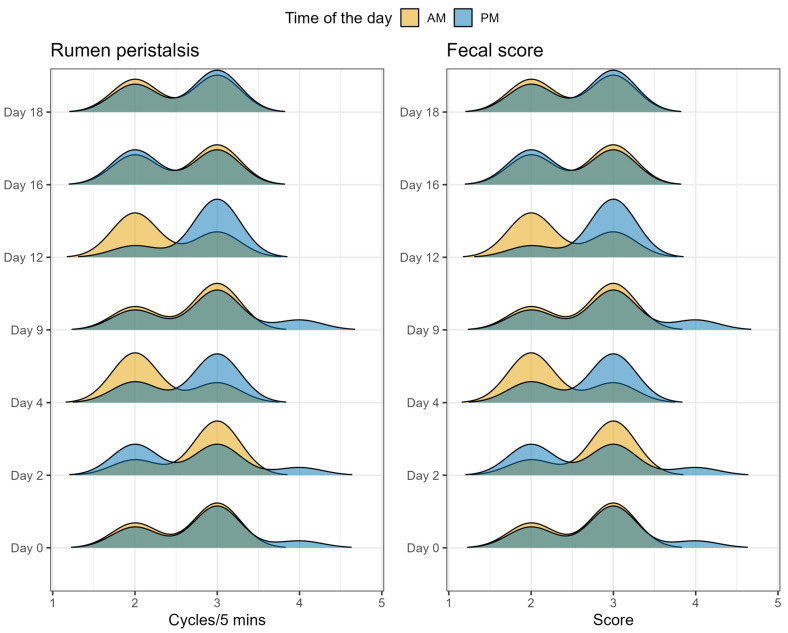
Effect of duration of exposure to ZEN (9.45 mg/day) on rumen peristalsis and fecal score of lactating Simmental cows. Measurements were taken twice per day, after each feeding time in the morning (AM) and afternoon (PM). The duration of exposure to ZEN did not significantly impact rumen peristalsis and fecal score.

As reported in Figure 2, ZEN exposure increased body temperature on days 9, 16 and 18 compared to baseline (*p* < 0.01), while measuring time in the morning or afternoon did not influence this parameter. Similarly, heart rate and respiratory rate increased with length of exposure to ZEN. Heart rate increased on days 9 and 12 (*p* < 0.01); meanwhile, respiratory rate increased on days 2 and 4 (*p* < 0.01) compared to baseline. Additionally, respiratory rate decreased on day 16 (*p* < 0.01) compared to baseline. Heart rate increased for the PM sampling on days 4, 9, and 18, with *p* < 0.01, 0.05, and 0.01, respectively. Respiratory rate followed the same pattern, decreasing 7 h after first ZEN exposure on day 16 (*p* < 0.01). Furthermore, during the baseline, respiratory rate also increased 7 h after morning feeding (*p* < 0.05). Rumen peristalsis and fecal score did not change with length of ZEN exposure nor due to the time of day, as shown in Figure 3.

### 2.6. Blood Parameters

**Figure 4:**
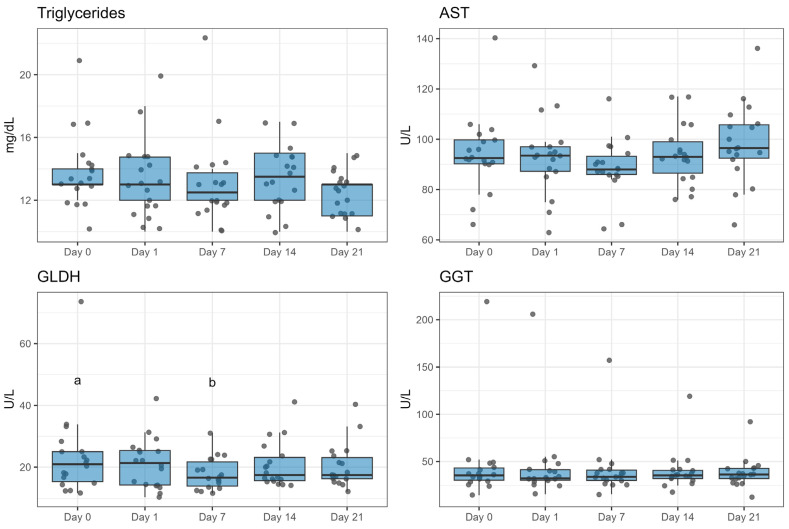
Effect of duration of exposure to ZEN (9.45 mg/day) on blood parameters related to liver health of lactating Simmental cows. AST: Aspartate aminotransferase; GLDH: Glutamate dehydrogenase; GGT: Gamma glutamyltransferase. Different letters (a, b) indicate a significant difference (*p* < 0.05) compared to week 0. The duration of exposure to ZEN had a significant effect on GLDH (*p* < 0.05) and tended to affect AST (*p* = 0.08).

Triglycerides and gamma glutamyltransferase (GGT) did not change with ZEN exposure. On the other hand, aspartate aminotransferase (AST) tended to increase from day 7 to day 21 (*p* = 0.06) and glutamate dehydrogenase (GLDH) decreased on day 7 compared to baseline (*p* < 0.05; Figure 4).

## 3. Discussion

In the literature, there is a knowledge gap in terms of controlled experiments evaluating the animal health response variables of lactating cows exposed to ZEN, since most of the old and recent publications focused on reproductive parameters [32,33,34,35]. This study aimed to evaluate a 3-week-length exposure to ZEN in lactating cows and its impact on animal health parameters, milk characteristics, rumen and fecal fermentation, and chewing activity. Supporting our hypothesis, certain systemic health parameters were negatively modulated with a daily intake of 9.45 mg ZEN, which is still below the EFSA recommendations. Specifically, heart rate and body temperature were the variables that were most negatively impacted, increasing as the length of ZEN exposure was prolonged to days 9, 12, 16, and 18. It is important to underline that health parameters such as temperature and respiration rate were evaluated considering the daily fluctuations in environmental temperature and diurnal patterns in the animal. Therefore, the reported effects can be ascribed to ZEN exposure and show signs of compromised health. Our research provides new knowledge on the effects of ZEN on systemic health, since most of the previous studies investigated the health parameters focusing on other mycotoxins or on a combination of different toxins. For instance, some reports exist in regard to aflatoxin exposure with no significant effects on multiparous Holstein cows fed 2.154 mg of aflatoxin B1 (AFB_1_), in terms of body temperature, heart and respiratory rates, and fecal score [36]. Some other research on different species such as dogs report that body temperature did not change but heart rate increased after ZEN and DON exposure [37]. A *Fusarium* toxin, DON, decreased body temperature in mice [38]. A similar trial evaluating ZEN alone reported similar results of increasing body temperature 10 h after exposure but no effects on the heart and respiratory rates of dry Holstein cows [22]. According to some literature, fecal consistency was more loose when cows were exposed to feedstuff contaminated with a mixture of mycotoxins, including ZEN [39], which is in contrast with our findings and may be due to the synergistic effect of the diverse mycotoxins.

Our findings contradict other reports evaluating ZEN and DON together [31,33] or a mixture of AFB_1_, Ochratoxin A, and ZEN, which showed decreased DMI [40]. Likewise, McKay et al. [41] demonstrated that a greater *Fusarium* contamination decreased DMI by 0.45 kg when exposed to 366 μg ZEN/kg DM TMR compared to a lower contamination level of 19 μg ZEN/kg DM TMR. Additionally, Winkler et al. [42] demonstrated no influence on DMI with fresh cows fed contaminated feed with DON and ZEN. Contradicting our hypothesis, ZEN intake did not negatively influence DMI, but in fact increased DMI with ongoing ZEN exposure. We expected a lower appetite due to the systemic consequences of chronic exposure to the toxin. Nevertheless, similar findings have been reported in the literature. For instance, Hartinger et al. [22] and Dänicke et al. [23] found tendencies towards a higher DMI when diets were contaminated with ZEN. It could be that the low level of inclusion of ZEN in our trial was not perceived by the cows.

In terms of chewing activity, the literature has shown contradictory findings in the limited experiments evaluating ZEN, DON, or ZEN + DON. DON contamination has been reported together with a reduction in ruminating time [43]; meanwhile, Hartinger et al. [22] reported a tendency of ruminating time and chewing index to increase, as well as increased chews per bolus compared to a baseline in dry cows exposed to 5.9 mg of ZEN, contradicting our results. However, the findings of Hartinger et al. [22] agree with ours regarding no changes in ZEN for eating and drinking times. The differences between our trial and previous studies might be explained by the duration of the experiments. ZEN can be decomposed in the rumen [18,44] within minutes to hours of ingestion; this time and the detoxification rate of ZEN seems to be relevant in modulating changes in ruminating time.

The literature has demonstrated that when cows are exposed to subacute ruminal acidosis, the level of degradation of mycotoxins is depleted [45]. Following the same pattern and with a similar dietary ration, Hartinger et al. [22] reported a decrease in rumen fluid pH in ZEN-exposed dry cows, thus contrasting with our findings. Similar findings to ours, including no changes in rumen pH, were reported by Dänicke et al. [46] for cows fed contaminated wheat. Our results on rumen fluid pH offer a limited picture and cannot fully represent daily variations since we had a single sampling point 1 h after the first morning exposure to ZEN. Nevertheless, this sampling point was chosen considering the fast action of rumen microbiota on the toxin. In fact, with an in vitro set-up, Gruber-Dorninger et al. [18] also reported a decrease in rumen fluid pH after 3 h of incubation and exposure to ZEN. The effects of ZEN on ruminal pH seem to be linked with the dosage, rather than the duration of exposure.

Research in terms of rumen fluid osmolality was not conducted prior to this publication; however, interesting findings performed by Korosteleva et al. [47,48] confirmed an increase in blood osmolality with the intake of *Fusarium* mycotoxins. Osmolality is an indicator of water balance and the increase in this parameter at the rumen level may indicate a certain disbalance, considering that we also did not notice changes in drinking gulps. Findings in other species in terms of ZEN accumulation in tissues, such as kidney, warrant further research towards the functions of detoxification and excretion of this organ [49,50,51] and the possible connection with water balance.

The results for rumen fermentation parameters coincide with previous reports by Hartinger et al. [22] after 7 days of baseline and over two consecutive days of ZEN exposure, the toxin exposure decreased the total SCFA in the rumen, similar to our findings on day 7 of exposure. Increasing isobutyrate proportions after exposure to ZEN were also reported by Seeling et al. [52] 3 h after morning feeding of a diet contaminated with *Fusarium*. Different results were reported by Dänicke et al. [46], with no changes in rumen fermentation after cows were fed with DON- and ZEN-contaminated feed. These contrasting results may be because Dänicke worked with dry cows with a DMI ranging between 4 and 12 kg; meanwhile, in this study, the intakes averaged 24 kg and due to the synergistic effect of both mycotoxins together.

Fecal fermentation research related to ZEN intake has not yet been reported, limiting the discussion of our results within this parameter. We noticed an opposite pattern of the one described in the rumen, with increasing total SCFA at day 21 and an increasing acetate-to-propionate ratio on days 1, 14, and 21 due to a greater acetate and lower propionate concentration on those exact days. It is important to point out that the fecal fermentation values reported in this experiment are within the normal scopes reported by others [53,54]. A possible explanation of the increase in total fecal SCFA on day 21 may be due to an increase in mucins in the hindgut, a parameter that we did not measure. Mucins are demonstrated to increase fermentation products in vitro, when evaluated with rumen fluid and a single forage substrate [55]. Nevertheless, different findings were reported, including a decrease in mucin production at the gastrointestinal level after DON exposure in monogastrics [56,57]. The animals received the same diet throughout the trial; therefore, the differences in SCFA are likely a result of altered microbial activity due to ZEN, both in the rumen and feces.

Altered SCFA production due to ZEN might also impact milk composition. The literature has demonstrated different results regarding mycotoxin exposure and its effects on milk characteristics, with only some of them confirming changes in milk composition [31,33,58]. Nevertheless, our results did not show major changes in the main milk components, which is in line with Dänicke et al. [46]. Similarly, no changes in milk fat, protein, and yield in response to feed contaminated with DON + ZEN were reported by McKay et al. [41]; in SCC and lactose by Korosteleva et al. [47]; and in fat to protein ratio by Winkler et al. [42] with different lengths of exposure, i.e., 28, 56, and 91 days, respectively. The possible explanation for the lack of effects on milk composition in our trial may be the fact that acetate and propionate, the main determinants of milk fat and milk yield, were not affected by ZEN in the rumen and only marginally in the hindgut. Thus, the low level of ZEN, which is below the recommended limit of EFSA, did not impact the milk performance of the cows. Coinciding with pioneering research by Prelusky et al. [59] showing no significant residual ZEN in milk or plasma in lactating cows. Nevertheless, implications on human health due to intake in food can include hormone-dependent health problems such as prostate, ovarian, or cervical cancer [5].

Thus far, research focused on the effects of ZEN on the blood serum concentration of liver enzymes has also shown inconsistent results. Different publications working with ZEN and DON or DON alone have not demonstrated changes in liver enzyme concentrations, including AST and GGT [47,48,60], agreeing with our findings. Meanwhile, Gallo et al. [61], using feed contaminated with different *Fusarium* mycotoxins, reported increased values of AST and GGT. The different results could be explained perhaps due to the greater starch levels of the diets evaluated by Gallo in comparison with ours. The literature also shows us limited information for ZEN contaminations and their effects on liver-specific biomarkers, such as GLDH. Results thus far for different toxins are inconsistent as well. For example, a tendency towards an increase in GLDH was reported by Kinoshita et al. [60] when cows were fed DON. Meanwhile, an in vitro experiment conducted by Ramadoss and Mukherjee [62] using citrinin reported a decreased GLDH concentration in cows. Furthermore, the alterations of liver parameters might be connected to the increased body temperature of the animals, rather than with the hepatotoxicity of the mycotoxin [63,64,65].

## 4. Conclusions

Prolonged exposure to ZEN has negative implications on the health of lactating cows, even at levels below the EFSA guidance level (500 µg/kg 88% DM). Patterns of loss of water balance seem probable due to the increase in rumen osmolality. Furthermore, systemic health parameters like body temperature, respiratory rate, and heart rate were negatively modulated, with possible implications on liver functionality. However, DMI, rumination activity, and milk performance variables were not affected, which may occur due to the low level of ZEN inclusion. Rumen fermentation was initially affected after 7 days of exposure, but this change seemed reversed from day 14 to day 21, which may show the resilience of the rumen microbiome to ZEN exposure. Although the production parameters were not affected by the exposure to ZEN, the results obtained for osmolality and systemic health raise questions on the adequacy of the guidance levels in terms of animal welfare. More research regarding the evaluation of the implications of feed contaminated with ZEN for liver enzyme concentrations and inflammation markers is needed. It is also essential to consider future studies with high-yielding cows, which are typically fed high-concentrate diets and have a lower detoxification capacity that is more challenging with ZEN exposure.

## 5. Materials and Methods

### 5.1. Animals, Experimental Design and Management

The experiment included 18 lactating Simmental cows (4.61 ± 1.89 years, 139.61 ± 47.13 days in milk (DIM), mean lactation number of 2.78 ± 1.77, initial BW of 693.44 ± 47.13 kg, and producing 34.69 ± 6.19 kg of ECM at the start of the trial). The trial was conducted for 26 consecutive days and included 4 days of adaptation in which the cows learned to use the individual feed troughs, 1 day served as baseline measurements (day 0), while sampling days occurred on days 0, 1, 7, 14, and 21 for most of the evaluated parameters with the exception of systemic health parameters that were determined on days 0, 2, 4, 9, 12, 16, and 18.

Details of animal housing and TMR preparation are described by Hartinger et al. [22]. Water and feed were available for ad libitum consumption as well as free-choice mineral blocks. Cows received a TMR during the entire experiment, consisting of a 40% concentrate mixture (21% energy supplement, 19% protein supplement), 20% corn silage, and 40% grass silage (on DM basis); details about diet composition are reported in Table 4. The TMR was prepared daily, and fresh feed was offered twice per day (0800 and 1430 h). Each cow had an ear tag as an identification sensor allowing access to individual feed troughs, which were equipped with electronic weighing scales and computer-regulated access gates (Insentec B. V., Marknesse, The Netherlands); feeding troughs were checked daily before discarding the refusals to check for DMI. Individual feed intake was automatically recorded throughout the day. To ensure total contaminated TMR intake at every feeding time (morning and afternoon), each cow received 250 g of wheat grain + 4.6 mg ZEN in its feed trough. This addition was consistently mixed with ~2.8 kg DM TMR, and fresh uncontaminated TMR was added once the cows consumed the contaminated feed. The calculation of ZEN supplementation was based on an estimated DMI of 23 kg, targeting a daily ZEN intake of 400 µg/kg and aiming to maintain the levels below the maximum daily dose recommended by the European Commission [14]. Additional analyses for ZEN contamination of the TMR were performed in an external laboratory (Romer Labs Diagnostic GmbH, Getzersdorf, Austria) to ensure that the intake was still below the recommended limits using a multimycotoxin extended method with HPLC-MS/MS and isotopic labeled internal standard analysis. The TMR was sampled at the beginning and at the end of the experiment, and the ZEN concentration in the TMR ranged between 22.4 and 85.6 µg/kg. Additionally, α-ZOL and β-ZOL were below the limits of detection, at 10 and 6 µg/kg, respectively. The experiment was approved by the Ethics and Animal Welfare Committee of the University of Veterinary Medicine, Vienna, in accordance with the University’s Guidelines for Good Scientific Practice and authorized by the Austrian Federal Ministry of Education, Science and Research (ref 2023-0.062.024) in accordance with current legislation.

### 5.2. Sampling and Chemical Analysis of Feed

The TMR’s dry matter concentration was determined daily by drying samples at 100 °C for 24 h to adjust feed offered daily if needed. TMR samples were collected at the beginning and at the end of the trial for chemical composition and mycotoxin analysis. All nutrient analyses of feed samples were evaluated in duplicate according to the German Handbook of Agricultural Experimental and Analytical Methods (VDLUFA [67]). The DM, crude protein (CP), ether extracts (EE), NDF, ADF and ash were evaluated with different methods described in detail by Hartinger et al. [22]. Non-fiber carbohydrate content was calculated as 100 − (% CP + % NDF + % EE + % ash). Acid detergent lignin was gravimetrically determined after ADF separation with 72% sulfuric acid (method 6.5.3). Starch content was measured with the K-TSTA kit (Megazyme Ltd., Wircklow, Ireland).

Particle size distribution of the diet was determined according to Kononoff et al. [66]. With these data, physically effective NDF (peNDF) and the physically effectiveness factor (pef) were calculated following the methodology of Beauchemin and Yang [68]. The peNDF concentration of the diet was estimated with the multiplication of NDF content of the diet by its pef. The pef (ranged from 0 to 1) was calculated as the sum of the proportion of particles retained on the corresponding sieves (19.0 and 8.0 mm sieves for pef > 8 mm).

### 5.3. Monitoring of DMI and Chewing Activity

**Figure 5:**
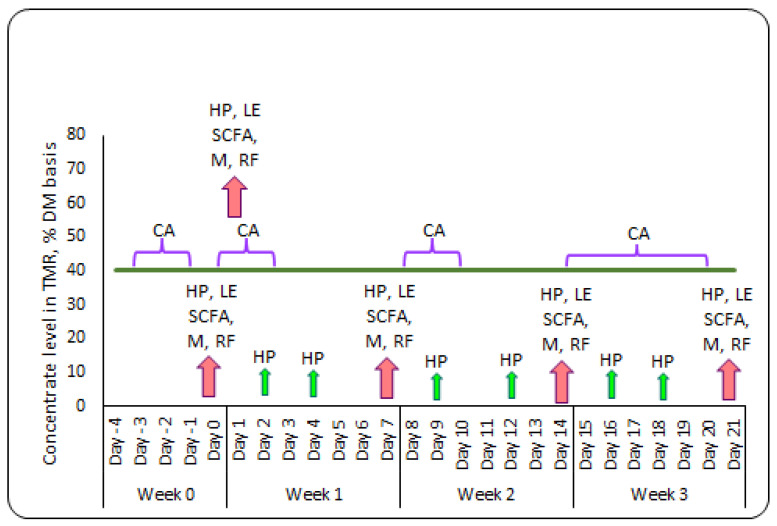
Sampling outline of the experimental period illustrating the sampling events. The purple braces indicate measurements conducted weekly, pink arrows indicate measurements conducted on days 0, 1, 7, 14, and 21, green arrows indicate health parameters. Acronyms: CA, chewing activity; HP, health parameters; LE, liver enzymes and tryglicerides; SCFA, short-chain fatty acid (rumen fluid and feces); M, milk yield and composition; RF, rumen fluid osmolality and pH.

The evaluation of chewing behavior was conducted weekly (Figure 5). This analysis included eating, ruminating, drinking time, number of chews per minute and per feed bolus, chewing index, drinking gulps, and total chewing time. More details about the methodology are described in detail by Kröger et al. [69] and Rivera-Chacon et al. [70]. For data transformation, we used the interface software RumiWatch Manager (version 2.2.0.0; Itin and Hoch GmbH), and we processed the data with RumiWatch Converter (Version 0.7.3.2).

### 5.4. Collection of Rumen Fluid for Osmolality, pH, SCFA, and Feces for SCFA Analysis

Rumen fluid sampling was performed 1 h after morning feeding. Approximately 80 mL of rumen fluid was collected using a stomach tube and then filtered using 4 layers of gauze. During animal collection, the stomach tube was rinsed with high-pressure water, while the jar where rumen fluid was collected was rinsed with water and disinfected with 70% ethanol. Aliquots were then transferred to 2 mL tubes and immediately frozen at −20 °C. At the end of experimental samplings, SCFA measurements were conducted following Qumar et al. [71].

Fecal samples were collected from cows’ rectums 9–10 h after morning feeding, transferred into 8 mL vials, and then immediately frozen at −20° C. Once at the laboratory, samples were thawed, and gas chromatography (GC-2010 PLUS, Shimadzu, Kyoto, Japan) was used to estimate SCFAs, including acetate, propionate, butyrate, valerate, isobutyrate, and isovalerate. After samples were thawed overnight at room temperature, subsamples of 1 g of feces were mixed with 1 mL of water, 300 µL of internal standard (4-methylvaleric acid, Sigma-Aldrich, St. Louis, MO, USA), and 200 µL of 25% phosphoric acid. After centrifugation at 20,000× *g* for 20 min at 4 °C, the supernatant was transferred into a fresh tube where the supernatant was again centrifuged at 20,000× *g* at 4 °C for 25 min, this step was repeated until the supernatant was clear. The clear solution was transferred into a GC vial and stored at −20° C until measurement. A 30 m × 0.53 mm ID × 0.53 µm df capillary column (Trace TR Wax, Thermo Fisher Scientific, Waltham, MA, USA) and a flame-ionization detector were used for separation and detection of SCFA. The injector and detector had temperatures of 220 °C, and helium was used as carrier gas with a flow rate of 6 mL/min. LabSolution LCGC software (version 5.97, Shimadzu) was used for the generation and evaluation of chromatograms.

### 5.5. Milk Sampling and Analysis

Cows were milked twice a day at 0630 and 1730 h in a 4 × 4 parallel tandem milking parlor (DeLaval GmbH, Eugendorf, Austria). Milk yield was registered by an electronic recorder (DeLaval Corp., Tumba, Sweden) daily during the entire length of the experiment. The ECM (kg/d) was determined: (0.38 × milk fat % + 0.21 × milk protein % + 0.95) × kg of milk/3.2 (GfE, 2001). Milk samples were taken from the morning and afternoon milking by in-line samplers on the day of baseline (d 0), and on days 1, 7, 14, and 21 to measure milk composition, and then stored in 50 mL tubes. Approximately 10–15 mL of the morning milking samples were stored with a conservation liquid (Eco Bronysolv GK145, ANA.LI.TIK. Vienna, Austria) and refrigerated at 4 °C until afternoon milking. The milk samples collected in the afternoon were added in approximately equal amounts to those of the morning, uniformly mixed, and stored at 4 °C until analyzed for fat, protein, lactose, MUN, SCC, and pH with infrared spectrophotometry (CombiFoss TM7, Foss, Hillerød, Denmark).

### 5.6. Measurement of Health Parameters and Fecal Score

Body temperature, respiratory rate, heart rate, rumen peristalsis, and fecal score were recorded on each sampling day, 1 h after morning and afternoon feeding (~0900 and 1530 h). Body temperature was measured rectally using a thermometer (VT 1831; microLife, Switzerland). The respiratory rate was visually observed (recording the movements of the right thorax for 1 min), and the heart rate was determined by palpation of the *Arteria caudalis mediana* for 1 min. The number of rumen contractions to assess peristalsis was evaluated with a stethoscope (Littmann Classic III, 3M, Seefeld, Germany) in a time frame of 2.5 min, and the value was multiplied by 2 to complete a cycle of 5 min. Fecal consistency was visually evaluated, ranging from 1 (very fluid feces) to 5 (extremely dry and segmented feces) following the methodology of Skidmore et al. [72].

### 5.7. Blood Sampling and Liver Enzyme Analysis

Blood samples were collected on days 0, 1, 7, 14 and 21,starting 1 h after morning feeding from the jugular vein, serum was sampled using 9 mL vacutainer tubes (Vacuette; Greiner Bio-One, Kremsmünster, Austria). Serum tubes were allowed to clot for 2 h at room temperature. Samples were centrifuged at 2000× *g* at 4 °C for 15 min, and serum was stored in 2 mL tubes (Sarstedt, AG, Wiener Neudorf, Austria) at −80 °C until analysis. Blood parameters, including triglycerides, AST, GLDH, and GGT were measured using a conventional large-scale analyzer for clinical chemistry at the laboratory of the Central Clinical Pathology Unit, University of Veterinary Medicine, Vienna, using the standard enzymatic colorimetric analyses for clinical chemistry (Cobas 6000/c501; Roche Diagnostics GmbH, Vienna, Austria). The intra-assay coefficient of variation was <5% for all blood variables.

### 5.8. Statistical Analysis

Data including DMI, chewing activity, rumen fluid pH, osmolality, rumen fluid and fecal SCFA, milk yield, milk composition, health, and blood parameters were analyzed using SAS (version 9.4; SAS Institute, Cary, NC, USA). For health parameters, temperature humidity index (THI) data were used as covariate and, when not significant, these data were included in the model as a random effect. Prior to any statistical analysis, data were checked for outliers using Cook’s D with a 0.08 threshold, which were removed. PROC UNIVARIATE was used to test normal distribution followed by the normal and plot options. When the normality assumption was not met, PROC TRANSREG performing a Box–Cox was used to determine the transformation mode (i.e., log or root square transformation), which was performed before the ANOVA. The statistical model had the fixed effects of day (duration of the ZEN exposure), parity, as well as time (for health parameters measured twice a day) and as the random effects cow and DIM. However, the data obtained from the same cow in different times were processed as repeated measures in the ANOVA, with a first-order variance–covariance structure matrix, considering that the covariance decays with time. Data are reported as LSM, and the transformed data were transformed back after the ANOVA. The largest standard error of the mean (SEM) was reported. Statistical significance was declared at *p* < 0.05 and had a tendency at 0.05 < *p* ≤ 0.10. Additionally, figures were created using R software (R Core Team, 2020) with ggplot2 package version 3.4.4 [73].

## Figures and Tables

**Table 1:** Effect of duration of exposure to ZEN (9.45 mg/day) on dry matter intake and chewing activity variables of lactating Simmental cows.

Item	Week 0	Week 1	Week 2	Week 3	SEM ^1^	*p*-Values ^2^
						Duration of Exposure
Dry matter intake, kg/day	22.25 ^b^	22.64	23.86 ^a^	23.59 ^a^	3.59	<0.01
Ruminating time, min/day	411.44	370.19	398.15	382.53	68.02	0.85
Ruminating chews/min	66.25 ^b^	66.98	66.82	67.77 ^a^	1.16	<0.01
Ruminating chews/bolus	57.74	59.72	60.09	57.44	2.09	0.55
Eating time, min/day	180.79	201.86	208.44	210.81	17.38	0.17
Drinking time *, min/da	6.92	8.64	7.14	7.79	2.61	0.24
Drinking gulps ^+^, No.	133.17	158.07	132.12	142.19	36.88	0.33
Total chewing time, min/day	642.55	666.73	683.31	671.74	25.29	0.59
Chewing index, time/kg DMI	27.47	29.37	28.14	27.89	1.39	0.51

^1^ The largest standard error of the mean; ^2^ *p*-Values for the effect of duration of exposure to ZEN in weeks. * Due to lack of normal distribution, values were first log transformed prior to statistical analysis. ^+^ Due to lack of normal distribution, values were first root square transformed prior to statistical analysis. ^a,b^ Means with different superscripts indicate a significant difference (*p* < 0.05) compared to week 0.

**Table 2:** Effect of duration of exposure to ZEN (9.45 mg/day) on rumen and fecal fermentation of lactating Simmental cows.

Item	Day 0	Day 1	Day 7	Day 14	Day 21	SEM ^1^	*p*-Values ^2^
							Duration of Exposure
Rumen fluid ^3^							
Total SCFA, mM	104.69 ^a^	99.75	83.37 ^b^	94.96	94.14	4.46	<0.01
% of total SCFA							
Acetate	59.01	58.04	58.58	58.39	58.45	2.45	0.41
Propionate	24.77	25.22	25.91	26.19	26.57	4.85	0.25
Butyrate	10.58	11.09	10.21	10.34	9.82	0.33	<0.05
Isobutyrate	0.89 ^ax^	0.90	0.80 ^y^	0.77 ^b^	0.78 ^b^	0.03	<0.01
Valerate	1.72 ^x^	1.74	1.63	1.59 ^y^	1.60 ^y^	0.05	<0.01
Isovalerate	1.28	1.33	1.30	1.14	1.19	0.21	<0.05
Caproate	0.80 ^ax^	0.76	0.63 ^b^	0.66 ^y^	0.66	0.07	<0.05
Heptanoate	0.12	0.09	0.09	0.10	0.10	0.15	0.73
Acetate:propionate ratio	2.49	2.41	2.35	2.34	2.31	0.61	0.32
Feces ^4^							
Total SCFA, mM	46.13 ^b^	48.51	46.52	51.35	62.07 ^a^	4.93	<0.05
% of total SCFA							
Acetate	75.28 ^b^	77.24 ^a^	76.49	77.20 ^a^	77.84 ^a^	0.41	<0.01
Propionate	15.93 ^a^	14.55 ^b^	15.22	14.71 ^b^	14.48 ^b^	0.24	<0.01
Butyrate	6.07	5.94	5.94	6.18	5.67	0.26	0.27
Isobutyrate	1.07 ^a^	0.85 ^b^	0.89	0.68 ^b^	0.75 ^b^	0.06	<0.01
Valerate	1.10 ^a^	0.98	0.97 ^b^	0.88 ^b^	0.85 ^b^	0.04	<0.01
Isovalerate	0.53 ^a^	0.41	0.46	0.33 ^b^	0.37	0.05	<0.05
Acetate:propionate ratio	4.75 ^b^	5.34 ^a^	5.05	5.30 ^a^	5.40 ^a^	0.30	<0.01

^1^ The largest standard error of the mean. ^2^ *p*-Values for the effect of duration of exposure to ZEN in days. ^3^ Rumen fluid samples were collected 1 h after cows received ZEN. ^4^ Fecal samples were collected 9–10 h after cows received ZEN. ^a,b^ Means with different superscripts indicate a significant difference (*p* < 0.05) compared to day 0. ^x,y^ Means with different superscripts indicate a tendency for significant difference (0.05 < *p* ≤ 0.10) compared to day 0.

**Table 3:** Effect of duration of exposure to ZEN (9.45 mg/day) on milk yield and composition of lactating Simmental cows.

Item	Day 0	Day 1	Day 7	Day 14	Day 21	SEM ^1^	*p*-Values ^2^
							Duration of Exposure
Milk yield, kg/day	36.04	36.11	35.97	36.01	36.72	10.26	0.82
Energy-corrected milk, kg/day	35.60	35.34	34.37	36.35	36.64	1.43	0.12
Milk composition							
Fat, %	3.99	3.95	3.70	4.01	3.94	1.28	0.16
Protein, %	3.38	3.31	3.42	3.54	3.57	0.46	0.18
Lactose, %	4.92	4.97	4.90	4.96	4.97	0.17	0.29
Milk urea nitrogen, mg/dL	23.63 ^b^	21.83	27.90 ^a^	22.78	22.77	7.14	<0.01
Somatic cell count *, cells/mL × 10^3^	34.88	34.37	33.07	35.74	33.03	1.21	0.86
Fat–protein ratio	1.12	1.13	1.02	1.07	1.05	0.04	<0.05
Milk pH	6.62 ^b^	6.61	6.57 ^b^	6.60	6.66 ^a^	0.14	<0.01

^1^ The largest standard error of the mean. ^2^ *p*-Values for the effect of duration of exposure to ZEN in days. * Due to lack of normal distribution, values were first log transformed prior to statistical analysis. ^a,b^ Means with different superscripts indicate a significant difference (*p* < 0.05) compared to day 0.

**Table 4:** Ingredients, chemical composition, and particle size distribution of diets fed to cows during the experiment.

	Diet, % DM (Unless Otherwise Stated)
Item	TMR
Ingredients	
Grass silage	20
Corn silage	40
Energy supplement ^1^	21
Protein supplement ^2^	19
TMR chemical composition	
DM, % as fresh	45.36 ± 0.41
Crude protein	14.9 ± 1.46
Neutral detergent fiber	39.41 ± 0.26
Acid detergent fiber	25.58 ± 0.86
Starch	28.67 ± 1.30
Ether extract	2.37 ± 0.09
Non-fiber carbohydrates	37.11 ± 2.49
Ash	6.22 ± 0.85
Particle fraction (% retained) ^3^	
Long	9.95
Medium	56.20
Short	21.78
Fine	12.06
pef ^4^	0.67
pe NDF ^5^ > 8 mm	26.38

^1^ Rindastar SM VET Schaumann GmbH contained maize, barley, wheat, calcium carbonate, sodium chloride, magnesium oxide, monocalcium phosphate, sodium bicarbonate, a premix with vitamins, and trace elements. Chemical composition: crude protein (9.0%), fat (3.0%), crude fiber (2.8%), ash (9.5%), net energy of lactation (6.9 MJ), calcium (1.8%), phosphorus (0.36%), sodium (0.75%), and magnesium (0.47%). Vitamins: vitamin A (45,000 IU), vitamin D3 (9000 IU), vitamin E (150 mg). Trace elements: iodine (calcium iodate, anhydrous) 15 mg, cobalt (coated cobalt (II) carbonate granules) 2.0 mg, copper (copper (II) sulfate pentahydrate) 50 mg, copper (copper (II) glycine chelate hydrate) 30 mg, manganese (manganese (II) oxide) 164 mg, manganese (glycine manganese chelate, hydrate) 44 mg, zinc (zinc oxide) 250 mg, zinc (glycine zinc chelate, hydrate) 90 mg, selenium (sodium selenite) 2.4 mg; ^2^ Rindastar 39% XP Schaumann GmbH contained rapeseed meal, dried distiller grains with solubles, heat-extracted soybean meal, urea, and molasses. Chemical composition: crude protein (40.0%), fat (3.6%), crude fiber (9.5%), ash (8.0%), net energy of lactation (6.7 MJ), calcium (0.9%), phosphorus (1.0%), sodium (0.25%), and magnesium (0.5%). Vitamins: vitamin A (9000 IU), vitamin D3 (1800 IU), vitamin E (30 mg). Trace elements: iodine (calcium iodate, anhydrous) 2.9 mg, cobalt (coated cobalt (II) carbonate granules) 0.4 mg, copper (copper (II) sulfate, pentahydrate) 10 mg, copper; ^3^ Particle fractions determined with the Penn State Particle Separator with a 19 mm screen (long), 8 mm screen (medium), 1.18 mm screen (short), and a pan (fine), (Kononoff et al. [66]); ^4^ Physical effectiveness factor; ^5^ Physically effective NDF.

## Data Availability

Data are contained within the article.

## List of Abbreviations

ZEN, zearalenone; α-ZOL, α-Zearalenol; β-ZOL, β-Zearalenol; SCFA, short-chain fatty acid; DON, deoxynivalenol; DMI, dry matter intake; SEM, the largest standard error of the mean; mM, mol/L; ECM, energy-corrected milk; SCC, somatic cell count; MUN, milk urea nitrogen; GGT, gamma glutamyltransferase; AST, aspartate aminotransferase; GLDH, glutamate dehydrogenase; EFSA, European Food Safety Authority; AFB_1_, aflatoxin B1; TMR, total mixed ration; DIM, days in milk; HPLC-MS/MS, high-performance liquid chromatography and mass spectrometry; NDF, neutral detergent fiber; ADF, acid detergent fiber; pef, physical effectiveness factor; peNDF, physically effective NDF; CP, crude protein; EE, ether extracts; THI, temperature humidity index.
